# Acute-Onset Vitreous Hemorrhage of Unknown Origin before Vitrectomy: Causes and Prognosis

**DOI:** 10.1155/2015/429251

**Published:** 2015-10-04

**Authors:** Dong Yoon Kim, Soo Geun Joe, Seunghee Baek, June-Gone Kim, Young Hee Yoon, Joo Yong Lee

**Affiliations:** ^1^Department of Ophthalmology, College of Medicine, Chungbuk National University, Cheongju, Republic of Korea; ^2^Department of Ophthalmology, Gangneung Asan Hospital, University of Ulsan, College of Medicine, Gangneung, Republic of Korea; ^3^Department of Clinical Epidemiology and Biostatistics, Asan Medical Center, University of Ulsan, College of Medicine, Seoul, Republic of Korea; ^4^Department of Ophthalmology, Asan Medical Center, University of Ulsan, College of Medicine, 88 Olympic-ro 43-gil, Songpa-gu, Seoul 138-736, Republic of Korea

## Abstract

*Purpose*. To analyze causes and prognosis of acute-onset preoperatively unknown origin vitreous hemorrhage (VH). *Methods*. This study included patients who underwent vitrectomy for acute-onset preoperatively unknown origin VH. The underlying causes of VH, which were identified after vitrectomy, were analyzed. And overall visual prognosis of unknown origin VH was analyzed. Risk scoring system was developed to predict visual prognosis after vitrectomy. *Results*. 169 eyes were included. Among these, retinal vein occlusion (RVO), retinal break, and age-related macular degeneration (AMD) were identified in 74 (43.8%), 50 (29.6%), and 21 (12.4%) patients, respectively. After vitrectomy, logMAR BCVA significantly improved from 1.93 ± 0.59 to 0.47 ± 0.71. However, postoperative BCVA in AMD eyes were significantly poorer than others. Poor visual prognosis after vitrectomy was associated with old age, poor preoperative vision in both eyes, and drusen in the fellow eye. *Conclusions*. RVO, retinal break, and AMD are the most common causes of acute-onset preoperatively unknown origin VH and the most common causes of VH change with age. The visual prognosis of unknown origin VH is relatively good, except among AMD patients. Older patients with poor preoperative BCVA in both eyes and patients with AMD in the fellow eye are at a higher risk of poor visual prognosis following vitrectomy.

## 1. Introduction

The annual incidence of acute-onset vitreous hemorrhage (VH) in the general population is 7 cases per 100,000 persons [1]. The causes of VH include proliferative diabetic retinopathy (PDR), trauma, retinal break, proliferative retinopathy after retinal vein occlusion (RVO), and posterior vitreous detachment without retinal detachment [2–6]. It is important to determine the underlying cause of acute-onset VH because the natural history and visual prognosis depend on the underlying cause. The visual prognosis of VH caused by retinal break, posterior vitreous detachment, and branch RVO is relatively good [4, 6–10]. However, the visual prognosis of VH secondary to PDR and exudative age-related macular degeneration (AMD) is poor due to recurrent VH, tractional retinal detachment, and submacular hemorrhage [11–14].

VH caused by a retinal break may be less severe and resolve more rapidly. Therefore, upright head position and immobilization of the patient allow blood to settle down in the eye, eventually clearing VH [4, 10, 15]. In cases of VH caused by retinal neovascularization due to diabetic retinopathy or RVO, peripheral laser photocoagulation can regress the abnormal vessels, which may lead to VH.

However, in some cases, it is difficult to determine the underlying cause of acute-onset VH. Lean and Gregor reported that 14% of acute-onset VH eyes were diagnosed on follow-up examination and 4% remained undiagnosed after 1 year [4]. Little is known about the causes and visual prognosis of acute-onset VH of preoperatively unknown origin. Here, we analyzed the causes and overall visual prognosis of acute-onset VH of preoperatively unknown origin. We also analyzed the characteristics of the fellow eye in all patients. In addition, we generated a risk scoring system (RSS) to predict visual prognosis of acute-onset preoperatively unknown origin VH.

## 2. Materials and Methods

### 2.1. Study Design and Participants

This retrospective review was conducted on patients who underwent vitrectomy for acute-onset VH of preoperatively unknown origin at Asan Medical Center, Seoul, Republic of Korea, between January 2007 and June 2013. The following inclusion criteria were applied: (1) a history of 3-port pars plana vitrectomy for acute-onset VH, with or without cataract surgery, and (2) the cause of acute-onset VH could not be preoperatively identified because of dense VH which prevented retinal examination. Exclusion criteria included eyes with other ocular diseases that might affect vision, active intraocular inflammation and/or infection, and VH caused by trauma. Patients with more than mild nonproliferative diabetic retinopathy in the fellow eye and those with grades III–IV AMD (AREDS (age-related eye disease study) classification) in the fellow eye were also excluded. This study was approved by the institutional review board of Asan Medical Center and followed the tenets of the Declaration of Helsinki (2014-0358).

### 2.2. Primary and Secondary Objectives

The primary objective of this study was to analyze the causes of acute-onset VH of preoperatively unknown origin. The secondary objectives of this study were to determine (1) the causes of acute-onset VH of preoperatively unknown origin according to age, (2) the visual prognosis of acute-onset VH of preoperatively unknown origin, (3) differences in the baseline characteristics according to postoperative diagnosis, and (4) characteristics of the fellow eye and (5) to devise an RSS for predicting the visual prognosis of acute-onset VH of preoperatively unknown origin.

### 2.3. Ophthalmic Examinations

All included patients underwent a complete bilateral ophthalmic examination, including determination of BCVA using the Snellen chart. BCVA results were converted to the logMAR scale. Patients who were only able to count fingers, were only able to detect hand motion, had light perception, or had no light perception were assigned logMAR values 2.0, 2.3, 2.7, and 3.0, respectively [16]. All patients also underwent biomicroscopic examination, dilated fundus examination, and fundus photography of both eyes. Ultrasonography was performed on all eyes with VH of preoperatively unknown origin.

### 2.4. Identification of Causes of Acute-Onset VH of Preoperatively Unknown Origin

The postoperative diagnosis of acute-onset VH of preoperatively unknown origin was identified by thorough review of each patient's medical records. We divided all included patients according to age (≤60, 60–70, and >70 years), and the causes of VH were analyzed according to age.

### 2.5. Surgical Procedures

Vitrectomy was performed by well-experienced retinal surgeons (Joo Yong Lee, June-Gone Kim, and Young Hee Yoon). A 20-gauge, 23-gauge, and 25-gauge vitrectomy system was used to perform 3-port vitrectomy. Cataracts were extracted by phacoemulsification if the crystalline lens had significant opacity. After resolving the VH, which obscured retinal inspection, we attempted to determine the cause of VH and recorded the underlying cause of VH as an operative note. After completing vitrectomy, eyes with retinal break received perfluoropropane (C_3_F_8_) gas tamponade. For complicated cases, such as those with severe tractional retinal detachment or multiple retinal breaks, silicone oil tamponade was performed. The silicone oil was removed 6 months later.

### 2.6. Statistical Analysis

One-way ANOVA and the Bonferroni post test were used to analyze the visual prognosis of VH according to the postoperative diagnosis. According to the postoperative diagnosis, the clinical characteristics were analyzed using one-way ANOVA with the Bonferroni post test and Pearson Chi-square test. Finally, to develop an RSS for predicting visual prognosis of acute-onset VH of preoperatively unknown origin, multivariate logistic regression modeling was performed. Risk factors were selected using backward elimination from the full logistic model. Model discrimination was estimated using* C*-tatistic (or AUC), and calibration was assessed by determining agreement between the predicted and recorded prognosis of acute-onset VH of preoperatively unknown origin. We ran an internal validation of discrimination (*C*-statistic) to produce optimism-corrected values of the* C*-statistic by using bootstrapping with 500 replications of individuals that were sampled with replacement [17]. After considering the internally validated variables, we developed a scoring system using the model parameter estimates described by Sullivan et al. [18]. ROC curves from the original model and scoring system are presented. SPSS (version 21.0; SPSS, Inc., Chicago, IL) and R 3.0.2 (free software that can be downloaded at http://www.r-project.org/) with package “boot” and “pROC” were used to perform the statistical analyses.

## 3. Results

In total, 2031 eyes in 2031 patients underwent vitrectomy for VH at Asan Medical Center between January 2007 and June 2013. Among these 2031 patients, the underlying cause of VH was not identified in 169 eyes in 169 patients (8.3%), and these patients therefore satisfied the inclusion criteria for enrollment.

### 3.1. Primary Objective

The postoperative diagnoses of acute-onset VH of preoperatively unknown origin are listed in Table 1. RVO (74 eyes, 43.8%) was the most common cause. All RVO eyes had branch retinal vein occlusion (BRVO). There were no cases of central or hemiretinal vein occlusion. Among the 74 eyes with BRVO, foveal involvement was only found in 10 eyes (13.5%). Fifty (29.6%), 21 (12.4%), and 8 (4.7%) eyes were diagnosed with retinal break, wet age-related macular degeneration (wAMD), or retinal arterial macroaneurysm, respectively, as the cause of preoperatively unknown VH. In 12 eyes (7.1%), we could not identify any retinal pathology even after vitrectomy.

### 3.2. Secondary Objectives

**Figure 1:**
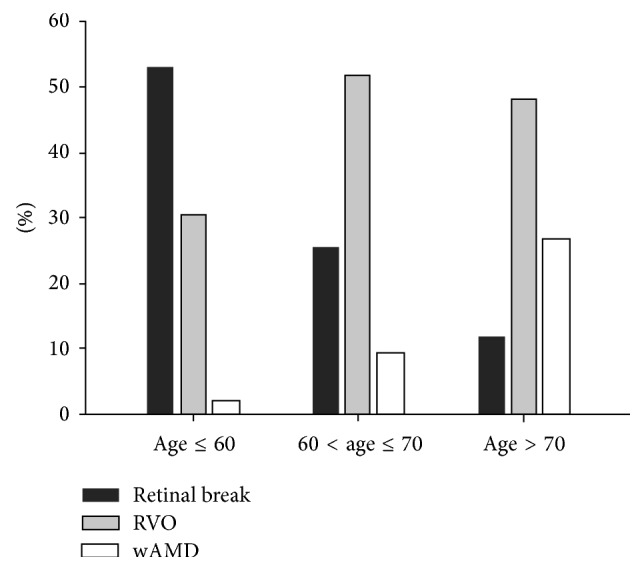
Causes of acute-onset vitreous hemorrhage of unknown origin according to age. Among patients aged ≤60 years, retinal break was the main cause of acute-onset VH of preoperatively unknown origin. Moreover, the proportion of cases of acute-onset VH of preoperatively unknown origin that was due to retinal break declined with age. In contrast to retinal break, the proportion of vitreous hemorrhage due to wAMD increased with age. Among patients aged >60 years, RVO was the most common cause of acute-onset VH of preoperatively unknown origin. RVO, retinal vein occlusion; wAMD, wet age-related macular degeneration.

**Figure 2:**
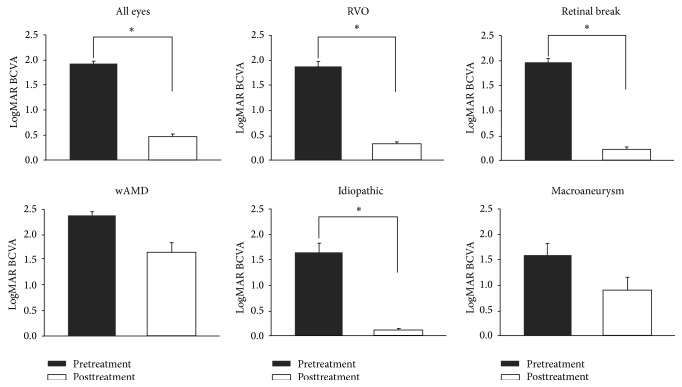
Changes in visual acuity after vitrectomy according to postoperative diagnosis. In the retinal vein occlusion, retinal break, and idiopathic groups, BCVA significantly improved after vitrectomy. However, visual improvement was not seen after vitrectomy among patients with wet age-related macular degeneration.

The causes of acute-onset VH of preoperatively unknown origin according to age are shown in Figure 1. We divided all included patients according to age (≤60, 60–70, and >70 years). In patients aged ≤60 years, retinal break was the most common cause of acute-onset VH of preoperatively unknown origin; however, the proportion of patients with retinal break declined with age. By contrast, the proportion of patients with wAMD increased with age. Among patients aged >60 years, RVO was the most common cause of acute-onset VH of preoperatively unknown origin. Visual prognoses according to postoperative diagnosis are shown in Figure 2. After vitrectomy, logMAR BCVA improved from 1.93 ± 0.59 to 0.47 ± 0.71. Preoperative logMAR BCVA significantly differed according to the postoperative diagnosis. The preoperative logMAR BCVA values of the wAMD patients were significantly poorer than those of other patients (wAMD, 2.37 ± 0.36; RVO, 1.87 ± 0.58; retinal break, 1.97 ± 0.57; *p* = 0.001). The postoperative logMAR BCVA values of the wAMD patients were also significantly worse than those of other patients (wAMD, 1.65 ± 0.88; RVO, 0.30 ± 0.43; retinal break, 0.21 ± 0.40; *p* < 0.001). Visual acuity changes after vitrectomy according to postoperative diagnosis are shown in Figure 2. BCVA significantly improved after vitrectomy among the RVO, retinal break, and idiopathic patients. However, vision did not improve after vitrectomy in wAMD patients.

The clinical characteristics of VH according to postoperative diagnoses are listed in Table 2. Mean age significantly differed according to the postoperative diagnosis. The mean age of the wAMD patients was significantly higher than that of the patients with RVO or retinal break (wAMD, 73.29 ± 8.30 years; RVO, 65.36 ± 9.82 years; retinal break, 58.50 ± 11.78 years; *p* = 0.001). Systemic hypertension was more frequently associated with RVO and wAMD patients. The characteristics of the fellow eyes among patients with acute-onset VH of preoperatively unknown origin are shown in Table 3. The logMAR BCVA values of the fellow eyes in wAMD patients were also significantly lower than in other patients (wAMD, 0.44 ± 0.45; RVO, 0.17 ± 0.38; retinal break, 0.05 ± 0.09; *p* < 0.001). Drusen was more significant in the fellow eyes of wAMD patients than in those of patients with RVO or retinal break (RVO, 6.8%; wAMD, 71.4%; retinal break, 2.0%; *p* < 0.001).

**Figure 3:**
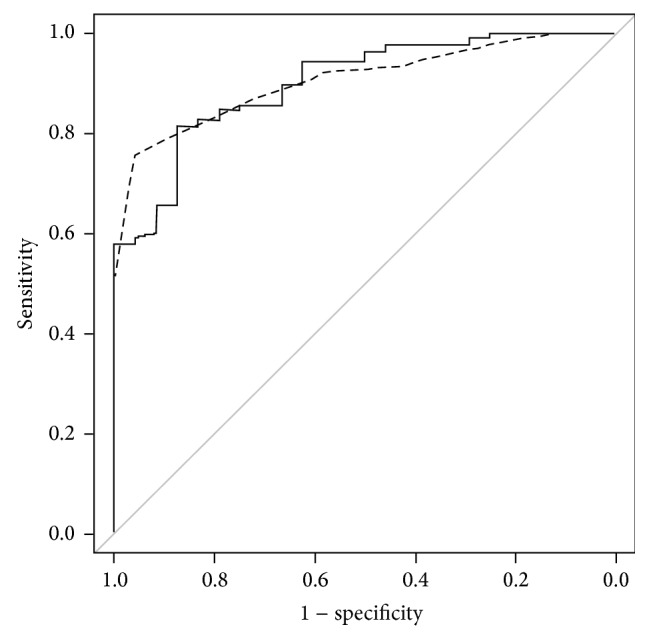
ROC curves of the scoring system used to predict the visual prognosis of acute-onset VH of preoperatively unknown origin. The solid line shows the ROC curves of four variables (age, baseline BCVA of the affected eye, baseline BCVA of the unaffected eye, and age-related macular changes in the fellow eyes), which were significantly associated with poor visual acuity following vitrectomy. The dashed line shows the ROC curve of the scoring system. The AUC of the scoring system was 0.907 (95% CI = 0.853–0.948).

The RSSs used to predict poor visual outcomes after vitrectomy among patients with acute-onset preoperatively unknown origin VH are shown in Table 4, and the risk scores of the included acute-onset VH of preoperatively unknown origin patients are shown in Table 5. The multivariate logistic regression model shows that old age, poor preoperative visual acuity in both the affected and fellow eyes, and drusen in the fellow eye were significantly associated with a poor visual prognosis after vitrectomy. The AUC of the scoring system was 0.907 (95% confidence interval [CI] = 0.853–0.948). Internal validation was investigated using the bootstrap validation algorithm. The optimism-corrected AUC was 0.898, which indicates the reliability of the RSS (Figure 3). In this RSS model, the maximal summation of risk score was −25. Therefore, to prevent obtaining a negative integer for the risk score, we added 25 to each estimated risk score.

## 4. Discussion

The etiology of VH is diverse, and the cause of VH is identified at the initial visit in 32–79% of eyes with VH [4, 6]. Most cases of acute-onset VH are caused by PDR, trauma, retinal break, proliferative retinopathy after RVO, or posterior vitreous detachment without retinal detachment [2–6]. However, it is often difficult to determine the underlying causes of acute-onset VH because dense VH obscures retinal examination. Lean et al. reported that no diagnosis was made at initial presentation in 21.0% of VH eyes, and Lindgren et al. reported that the underlying disease could not be determined in 68% of VH eyes [2, 4, 6, 19, 20]. Little is known about the causes and visual prognosis of preoperatively unknown origin VH. Therefore, in this study, we analyzed the causes and visual prognosis of acute-onset VH of preoperatively unknown origin.

We excluded VH cases that were associated with PDR. Diabetic retinopathy was assumed to be the cause of acute-onset VH of preoperatively unknown origin if the patient had a medical history of diabetes and fellow eye findings of PDR or severe nonproliferative diabetic retinopathy (NPDR). Therefore, patients with more than mild NPDR in the fellow eye were excluded. After excluding diabetic retinopathy, BRVO was the most common cause of acute-onset VH of preoperatively unknown origin VH (74 eyes [43.8%]).

According to previously published studies, except for PDR, retinal break is the most common cause of acute-onset VH [2, 4, 6, 19, 20]. Unlike previous studies, retinal break was the second most common cause of acute-onset VH of preoperatively unknown origin. This discrepancy between results can be explained by the characteristics of VH caused by retinal breaks. VH caused by a retinal break might be less severe and clear more rapidly [10]. Therefore, retinal breaks that lead to acute-onset VH can be found without vitrectomy. As a result, although retinal break may be the most common cause of acute-onset VH, the proportion of cases for which retinal break is the cause of acute-onset VH of preoperatively unknown origin was relatively small.

In the current study, we analyzed the visual prognosis after vitrectomy of acute-onset VH of preoperatively unknown origin. The overall visual prognosis of unknown origin VH was relatively good. Following vitrectomy, the logMAR BCVA improved from 1.93 ± 0.59 to 0.47 ± 0.71. However, the visual prognosis differed according to the underlying cause of preoperatively unknown origin VH. The preoperative and postoperative logMAR BCVA values of wAMD patients were significantly worse than those of patients with retinal break or RVO.

We believe that these differences in visual prognosis according to cause resulted from the different rates of foveal involvement. In our present study, only 10 cases (13.5%) of RVO involved the fovea and caused VH. In cases of RVO involving the fovea, patients were already diagnosed with RVO before the formation of retinal neovascularization, which eventually leads to VH. Therefore, the rate of foveal involvement was relatively small as a cause of acute-onset VH of preoperatively unknown origin. Because of the lower rate of foveal involvement, the visual prognosis of RVO as cause of acute-onset VH of preoperatively unknown origin was relatively good. By contrast, among wAMD patients with acute-onset VH of preoperatively unknown origin, the visual prognosis was poor due to subretinal disciform scar formation.

We also developed an RSS to predict the visual prognosis of unknown origin VH. Our multivariate logistic regression model showed that (1) old age, (2) poor preoperative visual acuity in the affected eye, (3) poor preoperative visual acuity in the fellow eye, and (4) drusen in the fellow eye are significantly associated with poor visual prognosis after vitrectomy. The sensitivity and specificity values of this scoring system were 0.88 and 0.71, respectively. With this simple RSS, we could predict the visual prognosis of acute-onset preoperatively unknown origin VH following vitrectomy. Because of the retrospective nature of this study, we could not validate the accuracy of this RSS. We therefore plan to conduct a future prospective study to validate this system.

To the best of our knowledge, this is the first study to analyze the causes and visual prognosis of acute-onset VH of preoperatively unknown origin. However, our analyses had limitations that are inherent to its retrospective design. In addition, this study was conducted at a single tertiary referring center, which might have caused some selection bias. The sample size of this study was also relatively small, which may have limited the statistical strength of the analysis. Therefore, future studies that examine a larger number of patients are needed to confirm the causes and visual prognosis of acute-onset VH.

In conclusion, the first, second, and third most common causes of acute-onset VH of preoperatively unknown origin were RVO, retinal break, and wAMD, respectively. In addition, the most common cause of acute-onset VH of preoperatively unknown origin changed according to patient age. The visual prognosis of unknown origin VH was relatively good, except among AMD patients. After considering age, preoperative BCVA, and the characteristics of the fellow eye, we predicted visual prognosis of unknown VH. Furthermore, the characteristics of patients with poor visual outcomes following vitrectomy include (1) old age, (2) low preoperative visual acuity in the affected and fellow eyes, and (3) age-related macular changes in the fellow eye. Therefore, in these patients, we expect visual prognosis of acute-onset VH of preoperatively unknown origin to be poor.

## Figures and Tables

**Table 1:** Causes of acute-onset vitreous hemorrhage (VH) of preoperatively unknown origin.

Underlying causes of VH	Eyes, *n* (%)
Retinal vein occlusion	74 (43.8%)
Central retinal vein occlusion (CRVO)	0/74 (0.0%)
Branch retinal vein occlusion (BRVO)	74/74 (100.0%)
BRVO with foveal involvement	10/74 (13.5%)
Vitreous hemorrhage with retinal break	50 (29.6%)
Age-related macular degeneration	21 (12.4%)
Macroaneurysm	8 (4.7%)
Eales disease	3 (1.8%)
Coats' disease	1 (0.6%)
Idiopathic	12 (7.1%)
Total	169 (100%)

**Table 2:** Clinical characteristics of acute-onset vitreous hemorrhage (VH) of preoperatively unknown origin.

	All	RVO	wAMD	Retinal break	Other	*p*
Number of eyes	169	77	21	50	24	0.001
Age (y)	63.64 ± 12.37	65.36 ± 9.82	73.29 ± 8.30	58.50 ± 11.78	60.63 ± 17.14	<0.001^*∗*^
Sex (male/female)	83/86	33/41	11/10	31/19	8/16	0.216^†^
Right/left	88/81	38/36	9/12	29/21	12/12	0.093^†^
Follow-up duration (mo.)	13.24 ± 14.52	11.34 ± 11.59	15.62 ± 19.19	15.56 ± 17.00	12.21 ± 12.49	0.360^*∗*^
Gauge of surgery						0.098^†^
20-gauge	19/169 (11.2%)	10/74 (13.5%)	5/21 (23.8%)	3/50 (6.0%)	1/24 (4.2%)	
23/25-gauge	150/169 (88.8%)	64/74 (86.5%)	16/21 (76.2%)	47/50 (94.0%)	23/24 (95.8%)	
Tamponade						<0.001^†^
No tamponade	56/169 (33.1%)	34/74 (45.9%)	4/21 (19.0%)	9/50 (18.0%)	9/24 (37.5%)	
Air	55/169 (32.5%)	32/74 (43.2%)	1/21 (4.8%)	13/50 (26.0%)	9/24 (37.5%)	
C_3_F_8_	24/169 (14.2%)	3/74 (4.1%)	1/21 (4.8%)	19/50 (38.0%)	1/24 (4.2%)	
Silicone oil	34/169 (20.1%)	5/74 (6.8%)	15/21 (71.4%)	9/50 (18.0%)	5/24 (20.8%)	
Systemic disease						
Hypertension	92/169 (54.4%)	48/74 (64.9%)	13/21 (61.9%)	17/50 (34.0%)	14/24 (54.4%)	0.006^†^
Duration (y)	8.44 ± 7.85	8.55 ± 8.46	6.92 ± 5.39	6.90 ± 5.52	11.57 ± 9.76	0.329^*∗*^
Diabetes	20/169 (11.8%)	9/74 (12.2%)	2/21 (9.5%)	7/50 (14/0%)	2/24 (8.3%)	0.891^†^
Duration (y)	9.56 ± 8.46	7.56 ± 4.95	6.50 ± 4.95	12.71 ± 4.45	13.44 ± 9.50	0.658^*∗*^
Anticoagulant	51/169 (30.2%)	34/74 (45.9%)	3/21 (14.3%)	8/50 (16.0%)	6/24 (25.0%)	0.001^†^

RVO, retinal vein occlusion; wAMD, wet age-related macular degeneration.

^*∗*^According to one-way ANOVA with the Bonferroni post test.

^†^According to the Chi-square test.

**Table 3:** Findings in the fellow eye of acute-onset vitreous hemorrhage (VH) of preoperatively unknown origin.

	All	RVO	wAMD	Retinal break	*p*
LogMAR BCVA	0.19 ± 0.46	0.17 ± 0.38	0.44 ± 0.45	0.05 ± 0.09	<0.001^*∗*^
Fundus finding					
Normal	131/169 (77.5%)	61/74 (82.4%)	4/21 (19.0%)	45/50 (90.0%)	<0.001^†^
Drusen	21/169 (12.4%)	5/74 (6.8%)	15/21 (71.4%)	1/50 (2.0%)	<0.001^†^
Retinal break	5/169 (3.0%)	0/74 (0.0%)	0/21 (0.0%)	4/50 (8.0%)	0.020
RVO	1/169 (0.6%)	3/74 (4.1%)	1/21 (4.8%)	0/50 (0.0%)	0.334
Others	8/169 (4.7%)	5/74 (6.8%)	1/21 (4.8%)	0/50 (0.0%)	0.177

BCVA, best-corrected visual acuity; RVO, retinal vein occlusion; wAMD, wet age-related macular degeneration; AMD, age-related macular degeneration.

^*∗*^According to one-way ANOVA with the Bonferroni post test.

^†^According to the Chi-square test.

**Table 4:** Risk scoring system used to predict poor visual outcomes after vitrectomy in patients with acute-onset vitreous hemorrhage (VH) of preoperatively unknown origin.

Categories	Reference value (*W*)	Beta	Beta(*W* − *W*_ref_)	Point = beta(*W* − *W*_ref_)/*B* [Clarify]	Score
Age					
24–40	33.5 (*W*_ref_)	−0.004	0	0	0
40–49	44.5		−0.043	−1.0000854	−1
50–59	54.5		−0.082	−1.9092539	−2
60–69	64.5		−0.121	−2.8184224	−3
>70	78.5		−0.176	−4.0912583	−4
Preoperative LogMAR BCVA in affected eye					
0–0.5	0.25 (*W*_ref_)	−0.112	0	0	0
0.5–1.0	0.75		−0.056	−1.3076873	−1
1.0–1.5	1.25		−0.112	−2.6153746	−3
1.5–2.0	1.75		−0.169	−3.9230619	−4
2.0–2.5	2.25		−0.225	−5.2307491	−5
2.5–3.0	2.75		−0.281	−6.5384364	−7
Preoperative LogMAR BCVA in the fellow eye					
0–0.5	0.25 (*W*_ref_)	−0.096	0	0	0
0.5–1.0	0.75		−0.048101477	−1.118639	−1
1.0–1.5	1.25		−0.096202955	−2.237278	−2
1.5–2.0	1.75		−0.144304432	−3.355917	−3
2.0–2.5	2.25		−0.19240591	−4.474556	−4
2.5–3.0	2.75		−0.240507387	−5.5931951	−6
Drusen in the fellow eye					
no	0 (*W*_ref_)	−0.358	0	0	0
yes	1		−0.358	−8.3255814	−8

BCVA, best-corrected visual acuity.

*W*: reference value.

Beta: beta coefficients used in the logistic regression model.

*B*: number of regression units that correspond to 1 point. We let *B* = 0.043 reflect the increase in risk associated with 10-year increases in age.

**Table 5:** Risk scores of study patients with acute-onset vitreous hemorrhage (VH) of preoperatively unknown origin.

Score^*∗*^	Estimated chance having good vision	All included patients	
		Snellen visual acuity < 20/200 after vitrectomy (no.)	Snellen visual acuity ≥ 20/200 after vitrectomy (no.)
0	0.448		
1	0.459		
2	0.470	1	0
3	0.480		
4	0.491	1	0
5	0.502	1	0
6	0.512	2	2
7	0.523	1	1
8	0.534	3	4
9	0.545	1	2
10	0.555	2	1
11	0.566		
12	0.576	2	1
13	0.587	0	1
14	0.597	1	2
15	0.608	2	4
16	0.618	6	17
17	0.628	1	37
18	0.638	0	29
19	0.648	0	19
20	0.657	0	9
21	0.667	0	5
22	0.677	0	6
23	0.686	0	3
24	0.695	0	2
25	0.704		
Total		24	145

^*∗*^Estimated risk score after 25 was added to prevent a negative integer as the risk score.
